# Impact of the COVID-19-Related Lockdown on Psychoactive Substance Consumption and Mental Health in Morocco

**DOI:** 10.4314/ejhs.v35i2.3

**Published:** 2025-03

**Authors:** Salma Ait Bouighoulidne, Amina Aquil, Maroua Guerroumi, Fatima zahra Laamiri, Abdeljalil Elgot

**Affiliations:** 1 Laboratory of Sciences and Health Technologies, Higher Institute of Health Sciences, Hassan First University, Settat, Morocco

**Keywords:** COVID-19, psychoactive substances, mental health, lockdown, Morocco, substance use patterns

## Abstract

**Background:**

The COVID-19 pandemic and its lockdown measures disrupted daily life globally, potentially impacting substance use and mental health. However, the effects in Morocco, shaped by cultural and social factors, remain underexplored. This study aimed to assess the impact of the COVID-19 lockdown on psychoactive substance use and mental health in Morocco, while identifying key socio-demographic determinants of these changes.

**Methods:**

A cross-sectional online survey was conducted in April 2020, with 1,001 participants who had used at least one psychoactive substance in the past year. Data on substance use changes, socio-demographic characteristics, and emotional states were collected. Chi-square and McNemar's tests were used for statistical analysis.

**Results:**

The results indicated a significant reduction in the consumption of tobacco, alcohol, cannabis, and other substances during the lockdown (p < 0.001). The main reasons for this decrease were health concerns (37.6%) and limited consumption opportunities (25.9%). Younger individuals (18-30 years), students, and those with lower incomes were more likely to reduce their use (p < 0.05). Furthermore, 71.3% of participants reported worsened emotional states, with worry (66.6%) and loneliness (52%) being the most common.

**Conclusion:**

The COVID-19 lockdown led to a significant reduction in substance use in Morocco, mainly due to restricted access and lifestyle changes. However, it was also associated with worsened mental health, highlighting the need for culturally appropriate mental health support and harm-reduction strategies during crises.

## Introduction

Psychoactive substances such as alcohol, tobacco, and cannabis are widely consumed globally, with usage patterns influenced by psychological stress, cultural norms, and social environments. While substance use does not always lead to addiction (1), even non-compulsive consumption can have significant implications for mental health, including increased anxiety, depression, and other psychological challenges (2). Thus, understanding the relationship between substance use and mental health requires a nuanced approach that distinguishes between occasional use, dependency, and broader psychological effects.

The COVID-19 pandemic disrupted daily life globally, with strict lockdown measures limiting social interactions, restricting movement, and potentially altering substance use behaviors (3). Previous research suggests that such disruptions can have complex effects on substance consumption. While some individuals increased their use as a coping mechanism for stress and uncertainty, others reported reductions or cessation due to financial constraints, reduced availability, or limited consumption opportunities (4–6). Additionally, changes in substance use patterns may influence mental health, although it remains challenging to determine whether psychological distress stems from altered consumption behaviors, the pandemic, or both. For example, studies have shown that alcohol, nicotine, and cannabis use increased among certain consumers who reported worsening mental health, compared to those who did not increase their consumption (7). In contrast, a study conducted in Iceland found that although depressive symptoms and the overall mental well-being of adolescents worsened during the COVID-19 pandemic beyond the expected increase based on previous trends, substance use actually decreased (8). This suggests that the relationship between mental health and substance use during the pandemic is complex and may vary across different populations and socio-cultural contexts.

In Morocco, cultural and religious norms strongly influence attitudes toward psychoactive substances. Islam, the dominant religion, strictly prohibits intoxicants, which shapes both societal perceptions and legal regulations surrounding substance use (9). These restrictions, combined with social stigma, may have played a key role in how individuals adapted their consumption behaviors during the lockdown. Gender differences are particularly pronounced in Morocco, where men generally have greater social mobility and access to substances, while women face stricter societal constraints (10). Despite these culturally specific dynamics, there is a notable lack of research on psychoactive substance use in Morocco, especially in the context of the COVID-19 pandemic.

This study aims to fill this gap by investigating how the COVID-19-related lockdown affected psychoactive substance consumption and mental health in Morocco. By examining socio-demographic factors, motivations for increased or reduced use, and associated emotional changes, this research provides critical insights for developing culturally tailored public health interventions. Furthermore, by situating Moroccan substance use behaviors within their religious and social context, this study contributes to a more comprehensive understanding of how pandemic-related restrictions influenced psychoactive substance use patterns in a conservative society.

## Materials and Methods

**Study design**: This cross-sectional study was conducted during the first COVID-19 lockdown in Morocco to examine how lockdown measures affected psychoactive substance consumption and mental health. By exploring socio-demographic factors, reasons for reduced substance use, and emotional changes, this research offers essential insights to inform culturally tailored public health interventions. Although not officially part of the Global Drug Survey (GDS), the study adapted selected questions from the GDS to the Moroccan context to ensure cultural and linguistic relevance. Recruitment began in April 2020, targeting participants nationwide through online platforms.

**Study size and recruitment**: The survey was disseminated via social networks, including Facebook, Instagram, and WhatsApp. Recruitment relied on private sharing by collaborators and targeted outreach to maximize national reach. However, regional representation may have been limited due to the online nature of the survey. Efforts included sharing the survey link across various networks and communication groups, but no paid advertisements were used. Participation was voluntary, and no financial compensation was provided. Upon accessing the survey link (created using Google Forms), participants were directed to an introductory page detailing the study's objectives, inclusion/exclusion criteria, and a consent form. Only those who provided informed consent could proceed to the questionnaire. After screening for completeness, a final sample of 1,001 participants was retained.

**Data collection**: The survey collected data on changes in substance consumption, socio-demographic characteristics, lifestyle modifications, and mental health during the lockdown period. The survey compared substance consumption patterns before and during the COVID-19 lockdown. Questions on substance use, adapted from the Global Drug Survey (GDS) and tailored to the Moroccan context, included:

1. Did you consume any substance in the year before the lockdown?
2. How often did you consume in the year before the lockdown?
3. Did you consume any substance during the lockdown?
4. How often did you consume during the lockdown?

Participants were also asked to report whether they experienced worsened emotional states during the lockdown. For each emotion (e.g., sadness, worry, loneliness), they were asked, “Do you feel [emotion] during the lockdown?” A “yes” response indicated the presence of that emotion, while a “no” response indicated its absence.

**Statistical methods**: Data analysis was conducted using SPSS version 20. The first part focused on descriptive results, while the second part explored correlations between the variables. The Chi-square test was employed to examine relationships between the independent qualitative variables, and McNemar's test was used to compare dependent qualitative variables. Independent variables included age, gender, city of residence, marital status, educational level, profession, community life, COVID-19 pandemic, and lockdown. Dependent variables included psychoactive substance consumption, frequency of consumption, place of consumption, and mental health among substance users.

**Ethical statement**: All procedures involving human participants in this study were conducted in accordance with the ethical standards set by the institutional and/or national research committees. Confidentiality and anonymity were ensured, in compliance with the Declaration of Helsinki and its subsequent amendments. This study is part of a project approved by the Moroccan National Center for Scientific and Technical Research (N°COV/2020/15).

## Results

**General data of the study population**: The sample consisted of 40.6% participants aged 18–25 years, with a mean age of 28.3 years (SD = 8.34). The sample was predominantly male (84%) with a male-to-female ratio of 5.26. Most participants resided in urban areas (92.7%) and were single (80.2%), with a high level of education (86.5%). Additionally, 46.3% were employed in the public sector. Nearly half (48.4%) of the participants reported earning 5,000 MAD or more, exceeding the national minimum wage (SMIG: 3,000 MAD) by approximately 66%, reflecting a relatively high socio-economic status. Furthermore, 75.9% of respondents reported living with their families, with 25.0% sharing a room with two other people (table 1).

**Table 1:** Study characteristics and sample

Variables	Population (N = 1001)	CI in 95%
Age range		
Under 18	10(1.0)	0,4 – 1,7
18-25	406(40,6)	37,3 – 43,6
26-30	342(34,2)	31,2 – 37,3
31-44	231(23.0)	20,6 – 25,5
45 and over	12(1,2)	0,6 – 1,9
Gender		
Male	841(84,0)	81,7 – 86,2
Female	160(16.0)	13,8 – 18,3
Place of residence		
Urban	928(92.7)	91.1 – 94.2
Semi-urban*	52(5.2)	3.9 – 6.6
Rural	21(2.1)	1.3 – 3.0
Marital status		
Single	803(80.2)	77.6 – 82.7
Married	148(14.8)	16.6 – 17.1
Divorced	47(4.7)	3.4 – 6.1
Widower	3(0.3)	0 – 0.7
Educational level		
Unschooled	3(0.3)	0 – 0.7
Literacy courses	14(1.4)	0.8 – 2.2
Secondary	118(11.8)	9.8 – 13.9
University	866(86.5)	84.3 – 88.5
Profession		
Student	187(18.7)	16.2 – 21.1
Public profession	463(46.3)	43.2 – 49.5
Liberal profession	254(25.3)	22.8 – 28.2
Unemployed	97(9.7)	7.9 – 11.7
Monthly income (MAD)		
<1000	186(18.5)	16.3 – 21.0
1000–1999	95(9.5)	7.7 – 11.4
2000–4999	236(23.5)	21.1 – 26.4
≥5000**	484(48.4)	45.1 – 51.3
Community life		
No	241(24.1)	21.5 – 26.8
Yes	760(75.9)	73.2 – 78.5

Note: The values are expressed in numbers and percentage.

* Semi-urban refers to areas with characteristics of both urban and rural environments, featuring moderate infrastructure development and service access.

** 5,000 MAD is approximately 500 USD

**Data related to psychoactive product consumption**: A comparison of psychoactive product consumption during the 12 months before the pandemic and during the lockdown revealed significant decreases in the usage of most products (p < 0.001). This included tobacco, alcohol, cannabis, hallucinogens, Kala, and chicha (p < 0.001 for most substances). Less than 20 people reported consuming solvents, cocaine, or other synthetic drugs during the lockdown (table 2).

**Table 2:** Consumption of psychoactive substances before and during the lockdown

Psychoactive Product	Population (N= 1001)		P

	12 months before the pandemic* declaration	During the pandemic period (lockdown)	
Tobacco			<0.001
No	200(20.0)	296(29.6)	
Yes	801(80.0)	705(70.4)	
Alcohol			<0.001
No	293(29.3)	655(65.4)	
Yes	708(70.7)	346(34.6)	
Cannabis			<0.001
No	538(53.7)	644(64.3)	
Yes	463(46.3)	357(35.7)	
Hallucinogens			<0.001
No	920(91.9)	989(98.8)	
Yes	81(8.1)	12(1.2)	
Chicha			0.002
No	978(97.7)	990(98.9)	
Yes	23(2.3)	11(1.1)	
Kala			<0.001
No	961(96.0)	978(97.7)	
Yes	40(4.0)	23(2.3)	
Cocaine			-
No	983(98.2)	(0)	
Yes	18(1.8)	(0)	
Synthetic Drugs**			-
No	986 (98.5%)	(0)	
Yes	15 (1.5%)	(0)	
Solvents			-
No	992 (99.1%)	(0)	
Yes	9 (0.9%)		

Note: The values are expressed in numbers and percentage. p value compared the consumption of psychoactive product before and during the covid-19 pandemic using the Mc Nemar test, p <0.05 is considered to be significant.

* The pre-pandemic period refers to the 12 months preceding the World Health Organization's declaration of the COVID-19 pandemic in March 2020.

** Synthetic drugs refer to psychoactive substances entirely created in laboratories, including amphetamines (e.g., MDMA), synthetic cannabinoids (e.g., Spice), and synthetic cathinones (e.g., ‘bath salts’). These substances have no natural base and are specifically designed to mimic or enhance the effects of natural drugs. Cocaine, on the other hand, is naturally derived from the coca plant and is presented separately from synthetic drugs due to differences in its origin and usage.

**Decrease in consumption and socio-demographic variables**: Data showed significant correlations between the decrease in psychoactive substance consumption and several socio-demographic variables, including age (p = 0.003), profession (p = 0.000), and income (p = 0.000). Younger adults (18–30) were more likely to reduce consumption, while adolescents and older adults showed minimal changes. Participants in public and liberal professions were less likely to reduce consumption compared to students and unemployed individuals. Additionally, those with higher incomes were less likely to decrease consumption (table 3).

**Table 3:** Correlation between the decrease in consumption and socio-demographic variables

Variables	Population N= 1001		P
		
	Decrease in use	No decrease	
Age range			0.003**
Under 18	10(1.3)	0(0)	
Between 18 and 25	331(43.3)	75(31.6)	
Between 25 and 30	252(33)	90(38)	
Between 30 and 45	163(21.3)	68(28.7)	
45 and over	8(1.1)	4(1.7)	
Gender			0.858*
Male	641(83.9)	200(84.4)	
Female	123(16.1)	37(15.6)	
Place of residence			0.947**
Urban	707(92.5)	221(93.2)	
Semi urban	40(5.2)	12(5.1)	
Rural	17(2.3)	4(1.7)	
Marital status			0.003**
Single	632(82.7)	171(72.2)	
Married	98(12.8)	50(21.1)	
Divorced	32(4.2)	15(6.3)	
Widower	2(0.3)	1(0.4)	
Educational level			0.980**
Unschooled	3(0.5)	0(0)	
Literacy courses	11(1.4)	3(1.3)	
Secondary	89(11.6)	29(12.2)	
University	661(86.5)	205(86.5)	
Profession			<0.001*
Student	161(21.1)	26(11.0)	
Public profession	331(43.3)	132(55.6)	
Liberal profession	192(25.1)	62(26.2)	
Unemployed	80(10.5)	17(7.2)	
Monthly income (MAD)			
<1000	162(21.2)	24(10.1)	<0.001*
Between 1000 and 2000	79(10.3)	16(6.8)	
Between 2000 and 5000	177(23.2)	59(24.9)	
>5000	346(45.3)	138(58.2)	

Note: The values are expressed in numbers and percentage, p <0.05 is considered to be significant.

* Chi-square test.

** Fisher test

**Impact of COVID-19 on mental health**: According to the survey, 71.3% of respondents experienced worsened emotions due to the pandemic and changes in their consumption patterns. The most prevalent emotions were worry (66.6%) and loneliness (52%) (Table 4).

**Table 4:** Distribution of the population according to emotional changes

Variables	Population (N=714)	CI in 95%
Worry		
No	240(33.6)	30.1 – 37
Yes	474(66.4)	63 – 69.9
Loneliness		
No	343(48.0)	44.3 – 51.8
Yes	371(52.0)	48.2 – 55.7
Anger		
No	373(52.2)	48 – 55.9
Yes	341(47.8)	44.1 – 52
Sadness		
No	378(52.9)	49.2 – 56.7
Yes	336(47.1)	43.3 – 50.8
Fear		
No	509(71.3)	67.9 – 74.5
Yes	205(28.7)	25.5 – 32.1
Pain		
No	614(86.0)	83.3 – 88.5
Yes	100(14.0)	11.5 – 16.7
Stigmatisation		
No	652(91.3)	89.2 – 93.3
Yes	62.87	6.7 – 10.8

Note: The values are expressed in numbers and percentage.

**Correlation between decreased consumption and emotional changes**: An analysis showed that emotional distress was significantly more pronounced among participants who reduced their consumption compared to those who did not reduce their consumption (78.7% vs. 21.3%, p = 0.005).

## Discussion

Substance use in Morocco is influenced by a complex interplay of cultural, religious, and legal factors. As a predominantly Muslim country, Morocco strongly discourages the consumption of psychoactive substances, especially alcohol and illicit drugs, due to religious prohibitions. Islam strictly forbids intoxicants, which shapes both societal attitudes and legal frameworks (9). Despite these restrictions, substance use persists, particularly among men who have greater social mobility and access to substances. This gender disparity is reflected in our findings, where men accounted for 84% of participants. The social acceptability of male substance use, combined with barriers limiting female participation, likely contributed to this imbalance. These findings contrast with those from other countries, where female participation in similar surveys was higher (11,12).

Our sample predominantly consisted of young, highly educated individuals, aligning with previous research that indicates a high prevalence of substance use among young populations (13) and individuals with higher education levels (14). However, this sample does not represent the broader Moroccan population, as many young adults, especially men, engage in family businesses or informal labor rather than higher education. The online survey method likely contributed to this selection bias, limiting the generalizability of our findings.

Although our study did not measure the frequency or severity of substance use, it is important to note that the majority of participants may have been occasional or social users rather than individuals with substance use disorders. Thus, while our results suggest a significant reduction in consumption during the lockdown, it likely reflects changes in access to substances and social environments rather than behavioral change or recovery.

The availability of psychoactive substances in Morocco is affected by legal status, regional differences, and enforcement policies. Alcohol is legally available but highly regulated, while cannabis has an underground market in certain regions (15). Regulatory factors, combined with pandemic restrictions, likely contributed to the reduction in substance use during the lockdown. However, this reduction does not necessarily indicate improved health outcomes, as many individuals may have reduced consumption out of necessity.

Our findings also underscore the emotional toll of the pandemic, as many participants reported worsened emotional well-being due to pandemic-related restrictions and changes in consumption. Similar findings have been observed in studies of other populations, indicating that increased substance use may have been a maladaptive coping mechanism for stress (7). However, a Moroccan study found that coping strategies like acceptance, positive reframing, and religious practices were more common, with substance use remaining infrequent (16).

This study's sampling method introduced bias, as the online survey mainly attracted a highly educated, urban population, limiting the generalizability of our findings to rural or lower-income groups. Additionally, the study did not measure the frequency or severity of substance use, which limits our ability to identify problematic use patterns. Finally, the study's descriptive nature prevents us from establishing causal relationships between COVID-19, substance use, and mental health outcomes.

In conclusion, our findings indicate that a significant proportion of young, educated Moroccans reduced their substance use during the lockdown, likely due to legal restrictions and limited access rather than voluntary behavioral change. The pandemic also negatively affected emotional well-being. However, the relationship between substance use and psychological distress remains complex, and future research should aim for more representative samples and longitudinal studies to assess whether reductions in substance use persist over time.
